# The Yin and Yang of VEGF and PEDF: Multifaceted Neurotrophic Factors and Their Potential in the Treatment of Parkinson’s Disease

**DOI:** 10.3390/ijms11082875

**Published:** 2010-08-05

**Authors:** Torsten Falk, Robert T. Gonzalez, Scott J. Sherman

**Affiliations:** Department of Neurology, University of Arizona College of Medicine, 1501 N. Campbell Avenue, Tucson AZ 85724-5023, USA; E-Mails: rtg@email.arizona.edu (R.T.G.); ssherman@u.arizona.edu (S.J.S.)

**Keywords:** neurodegeneration, VEGF-A, VEGF-B, PEDF, VEGFR1, VEGFR2, PEDF-R

## Abstract

Over the last few decades, vascular endothelial growth factor (VEGF) and pigment epithelium-derived factor (PEDF) have emerged as multifaceted players in not only the pathogenesis, but potential treatment, of numerous diseases. They activate diverse intracellular signaling cascades known to have extensive crosstalk, and have been best studied for their effects in cardiology and cancer biology. Recent work with the two factors indicates that the activity of one growth factor is often directly related to the action of the other. Their respective neuroprotective effects, in particular, raise important questions regarding the treatment of neurodegenerative disorders, including Parkinson’s disease.

## 1. Introduction

Current treatments for Parkinson’s disease (PD), while temporarily effective, are largely insufficient. Existing therapies fall short in their ability to treat the neuronal degeneration that underlies PD’s progressive nature. Over the last several decades, the growth factors VEGF and PEDF have become important subjects in research ranging from cancer therapy to the treatment of neurological disorders, including PD. Here we offer a brief overview of PD, examine in detail how VEGF and PEDF function individually and cooperatively, and explore how their neuroprotective effects may provide novel, more enduring approaches to the treatment of PD.

## 2. Parkinson’s Disease

The past decades have seen an unprecedented increase in research efforts in the interest of understanding and treating neurodegenerative disorders [1,2]. While this boost has certainly contributed to how we understand and treat neurodegenerative disorders, many treatments, including those for PD, remain fundamentally ineffective in modifying the course of the disease [1,3]. PD is the second most common neurodegenerative disorder affecting 2% of those age 65 years and older, and is characterized by loss of dopaminergic neurons of the substantia nigra [4]. The cause of PD remains unclear, but both environmental and genetic risk factors are likely [5,6]. For a minority of familial PD cases an association with specific genetic mutations is known [7]; these cases share both symptoms and treatment strategies with the 95% of PD patients with unknown etiology.

### 2.1. Onset and Symptoms

The presence of PD is typically identified by a number of hypokinetic motor symptoms that include: a slowing of physical movement (bradykinesia), tremor, rigidity, and postural instability. The most easily identifiable and common sign of PD is tremor. The resting tremor is often unilateral at the outset but involves both sides of the body as the disease progresses [8]. Another cardinal symptom of PD is bradykinesia, which, like rest tremor, is often asymmetric particularly in early stages, easily identifiable, and often noticed by the patient prior to neurological diagnosis. When combined with tremor, PD patients tend to have difficulty in tasks requiring fine motor movements and other actions that involve the planning and execution of movement. Though the cardinal movement symptoms are the most debilitating for most PD patients, there is a plethora of non-movement symptoms in PD that we will not discuss in detail, but include various changes in the autonomic nervous system, as well as neuropsychiatric symptoms that can occur in later stages of the disease [2].

### 2.2. A Critical and Timely Field of Research

Most medical treatments for PD rely on a dopamine replacement strategy and provide symptomatic relief only. These treatments are inadequate because they do not provide a continuous physiological level of dopamine and, moreover, fail to slow the progression of the underlying neurodegenerative processes. As a result, patients often develop disabling fluctuations in their motor symptoms as the disease progresses. These consist of periods of relative immobility when dopamine replacement levels are too low and the emergence of involuntary movements termed dyskinesia when dopamine exceeds normal physiological levels. For these reasons, much effort has been devoted to cell-based and gene-based neuroprotective strategies to either replace the lost neurons or prevent the progressive degeneration of the remaining dopaminergic neurons of the substantia nigra [9–11]. Both strategies have achieved some very promising results in animal models of PD as well as preliminary clinical trials, but unfortunately neither approach has been deemed effective in controlled clinical trials. Allogenic transplantation of fetal dopaminergic cells demonstrated successful engraftment and dopamine release [9,12]. Unfortunately, there was no significant clinical improvement and some patients developed dyskinesia even off of medication. Moreover, 2 recent papers reporting long term survival (>10 years) of fetal mesencephalic cell transplants showed α-synuclein inclusions in transplanted neurons of most patients, resembling Lewy bodies, the histopathological hallmark of PD, indicating that exogenous neuronal transplants are also vulnerable to the pathological processes that occur in PD [13,14]. A third study did not show this pathology in the postmortem analysis of five subjects [15].

Neurotrophic growth factors such as glial cell line-derived neurotrophic factor (GDNF) and brain-derived neurotrophic factor (BDNF), neurturin, fibroblast growth factor 2 (FGF-2) and others have shown great promise in experimental models of PD [16–18]. The hope is that using these factors in human disease could provide a potent disease-modifying therapy; however, clinical development of these neurotrophic agents is problematic. Monthly intracerebroventricular administration of GDNF delivery *via* a micro pump [17] and neurturin delivery *via* a viral vector-mediated gene transfer [ceregen-trial] ultimately failed in Phase II clinical trials. The disappointing results in clinical trials of neurotrophic factors despite robust preclinical data could be due to problems with the delivery method or the choice of neurotrophic factor. Improved delivery is currently the topic of many laboratories, and several new potential neurotrophic agents are being investigated. Thus, cell-based and gene-based neurotrophic or neurorestorative therapy remains an attractive approach but further research is necessary. In order for these disease-modifying therapies to be successful in the clinic, there is also a need for earlier diagnosis, since most dopaminergic neurons have already succumbed to the disease at the time of routine clinical diagnosis. It is encouraging that recent advances in imaging techniques [19], and a variety of other biomarkers [20,21] make earlier detection of PD a likely prospect for the near future.

The projected rise in the prevalence of PD and unprecedented economic effect on healthcare domestically and abroad makes further research into disease-modifying therapies a high priority amongst translational research. At the forefront of efforts to establish novel forms of treatment for PD and other neurodegenerative disorders is the use of neuroprotective agents aimed at addressing the underlying problems in neurodegenerative pathology.

## 3. Vascular Endothelial Growth Factor—A Versatile Growth Factor with Pathophysiological Implications and Therapeutic Potential

The polypeptide known as VEGF was first isolated in 1983 by Harold Dvorak and his colleagues, and was initially identified as vascular permeability factor (VPF) on account of its ability to induce vascular leakage [22]. In 1989, however, the same protein was purified by Napoleone Ferrara and noted for its role as a potent endothelial mitogen, for which it was essentially renamed “vascular endothelial growth factor,” or VEGF [23]. The protein Ferrara and his colleagues had purified was the most biologically active isoform of the VEGF family, VEGF-A, which is still commonly referred to simply as VEGF. This can be confusing at times, as a number of other related VEGF proteins have since been discovered. In this review, “VEGF-A” will be used to refer to VEGF-A_165_, the most biologically active and prototypically angiogenic isoform of the broader VEGF family. In the literature ‘VEGF’ is most of the time used synonymously for VEGF-A_165_. Other members of the VEGF family will be referred to specifically. This paper will primarily address research and history surrounding the VEGF-A isoform, but will also discuss particularly important findings relating to the neuroprotective properties of VEGF-B.

### 3.1. VEGF Isoforms and Structure

The VEGF family is comprised of five main members, namely VEGF-A, VEGF-B, VEGF-C, VEGF-D, and PLGF (placental growth factor) [23]. While VEGF-B, C, D, and PLGF serve various physiological roles, VEGF-A is generally regarded as the most biologically relevant member of the VEGF family [24]. The VEGF-A gene is divided among eight exons and seven introns, and alternative splicing of the gene’s RNA transcript gives rise to four different homodimeric VEGF-A isoforms: VEGF_121_, VEGF_165_, VEGF_189_ and VEGF_206_ [25], the subscript refers to the number of amino acid residues. Alternative splicing is a characteristic common to all VEGF isoforms with the exception of VEGF-C [26]. The largest of the four VEGF-A isoforms, VEGF_206_ and VEGF_189_ contain a highly basic amino-acid insertion, bind heparin with a high affinity, and are found tightly bound to cell surfaces or extra cellular matrix (ECM). VEGF_121_, in contrast, is acidic, does not bind heparin, and is a freely diffusible protein [27]. The larger three isoforms can undergo proteolysis by a variety of proteases, including plasmin, which has been found to cleave at Arg110-Ala111 yielding carboxy-terminal and amino-terminal fragments. Only the C-terminal fragment is shown to bind heparin, and loss of the C-terminal portion of the protein is associated with a significant decrease in VEGF’s mitogenic capacity [28]. VEGF_165_ exhibits properties similar to the other three species of VEGF-A in that it is capable of binding heparin and can be found either freely diffusible or sequestered in the ECM. The variable properties of VEGF_165_ are thought to contribute to its bioavailability and biochemical activity, as it is the most abundant and mitogenic of the four species [24].

### 3.2. VEGF Receptors and Co-Receptors

**Figure 1:**
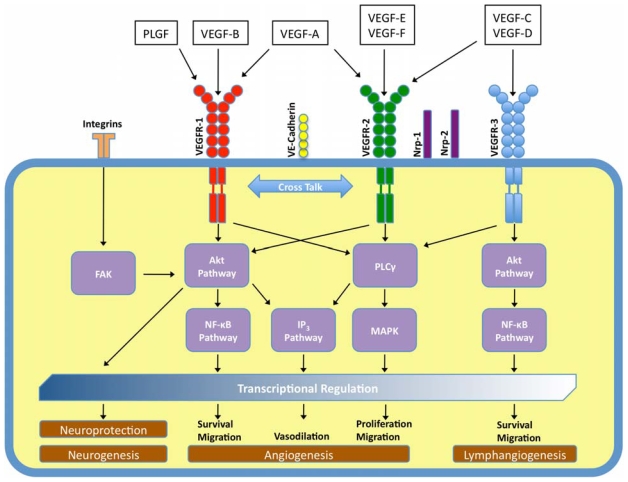
**Schematic of pathways activated by the different isoforms of the VEGF-family**. By binding to three receptors (VEGFR1–3) the different members of the VEGF-family of growth factors activate several intracellular signaling cascades that regulate a host of cellular functions. (FAK, focal adhesion kinase; Akt, also known as protein kinase B (PKB); PLCγ, phospholipase Cγ; IP_3_, inositol 1,4,5-trisphosphate; NFκB, nuclear factor κ-light-chain-enhancer of activated B cells; MAPK, mitogen-activated protein kinase).

The VEGF family is known to bind three different receptor tyrosine kinases (RTKs). As is typical of RTK activation, VEGF’s are dimeric glycoproteins and bind the RTK receptors crosslinking them as dimers. This causes the RTK’s cytoplasmic tyrosine kinase domains to autophosphorylate, triggering an intracellular signaling cascade that transduces the original signal of VEGF-binding into the cell, commonly *via* a MAP kinase. VEGF-A is known to bind two similar receptor RTKs, VEGFR1 or flt-1 (Kd ~10–20 pM) and VEGFR2 or flk-1 (~75–125 pM). VEGF-B binds to VEGFR1 only, whereas the other isoforms of VEGF are known to bind to VEGFR2 and VEGFR3 or flt-4 [29] (Figure 1). The VEGFRs can exist as homodimers or heterodimers.

VEGFR1 and VEGFR2 are both high affinity receptors for VEGF-A, and the structure of this subfamily is highly conserved, both in relation to one another and with respect to the RTK superfamily in general. All three receptors have an extracellular region comprised of seven immunoglobulin-like domains, a single transmembrane region, and an intracellular tyrosine kinase domain that is interrupted by a kinase-insert domain [30–32]. Studies have mapped the binding site for VEGF-A to the second immunoglobulin-like domain for each RTK, which likely forms hydrophobic interactions with the poles of the VEGF dimer that induce conformational changes across the transmembrane region, resulting in the intracellular signaling cascades mentioned above [33]. Experiments that involve the genetic deletion of VEGFR1 in mice have been shown to lead to embryonic lethality due to excessive vascularization [34], leading many to believe that VEGFR1 may regulate VEGFR2 activity by serving as a decoy receptor. This hypothesis has been corroborated further by experiments that reveal that genetic deletion of only VEGFR1’s tyrosine kinase domain do not result in embryonic lethality [35]. Further complicating the picture is that with VEGFR1 binding of its different ligands VEGF-A, VEGF-B and PLGF transduces distinct biological responses, and leads to different phosphorylation patterns [36].

In addition to the flt subfamily of RTKs, VEGF-A is known to interact with other cell-surface binding sites that serve as coreceptors to VEGF RTKs. These binding sites are referred to as neuropilins and exist in two known forms, both of which are known to interact with VEGF-A. The two forms of neuropilins are Neuropilin 1 and Neuropilin 2 (NRP-1 and NRP-2). Both neuropilins are transmembrane glycoproteins, but each lacks an intracellular kinase domain, and neither is believed to engage in any cellular signaling on its own [37]. That said, Soker *et al.* have characterized NRP-1 as an isoform-specific coreceptor capable of enhancing not only the binding of VEGF-A to VEGFR2, but VEGF-mediated cellular response to chemoattractants via chemotaxis [38]. VEGF-A is known to bind NRP-1 and NRP-2, while isoforms of PLGF and VEGF-B will only bind NRP-1.

### 3.3. VEGF Has Various Physiological Roles

Cellular homeostasis and survival is dependent upon a sufficient source of oxygen and nutrients, which in turn is dependent upon vascularization. Many pro-angiogenic growth factors are known to exist, but none are considered as potent or as predominant as VEGF-A. The discovery of VEGF-A had an enormous effect on our understanding of vascular proliferation, due to its prominent role in the vast majority of these processes. Through inhibition experiments and experimentally-induced gene mutations, VEGF-A has been shown to have a hand in everything from prenatal vasculogenesis to tissue regeneration in adults. Both normal and pathological actions of VEGF-A, including tumor proliferation and vascular endothelial cell permeability, are typically thought to occur via VEGFR2, despite the receptor’s lower affinity for VEGF-A relative to VEGFR1 [32,34]. For example, single allele deletions of VEGF-A have been shown to result in embryonic lethality in mice on account of angiogenic complications, including poor organ vascularization. The same results were observed when gene knockouts of the major receptors VEGFR1 or VEGFR2 were performed [39–41], whereas inactivation of VEGF-B and PLGF isoforms had no conspicuous phenotypic consequences. VEGF-A is just as crucial for neonatal development as for embryonic development. Inhibition of VEGF-A at this stage of development leads to an increase in mortality rate in neonatal mice due to a number of factors, including stunted body growth, impaired organ development, and renal failure [42].

VEGF-A’s functions in adult physiology are also of considerable importance, playing an indispensible part in diverse biological functions (Figure 1) such as ovarian angiogenesis, stem cell survival, and endochondral bone formation [24,43,44]. VEGF-A’s activity in adult vasculature was long thought to arise primarily in abnormal, pathogenic cases such as tumor angiogenesis, which have made it a promising target for cancer therapies [45,46]. Until recently, it was widely accepted that healthy adult vasculature was stable, and did not depend on VEGF-A for survival [42,47–49]. However, more recent studies show that VEGF-A plays an equally important role in the maintenance of pre-existing, established vessels in adults, including vascular beds in the adult intestine, thyroid, and liver [47,50]. Similarly, VEGF-B has been shown to be important for the survival of adult vasculature though dispensable for blood vessel growth. The survival effects extend to pericytes, smooth muscle cells, and vascular stem/progenitor cells [51].

Both VEGF-A and VEGF-B have been shown to have mitogenic effects on several cell types including hematopoietic cells [43,52], and there is also evidence suggesting a role for VEGF-A and VEGF-B in the regulation of adult neurogenesis [53,54]. Knowledge of the physiological role of VEGF-B and its isoforms is lacking, but it is known that it differs from other VEGF isoforms in its strongly decreased angiogenic capacity. This feature, in particular, has made it an intriguing subject for research concerning neurodegenerative disorders, the implications of which are discussed below.

### 3.4. Pathological Roles

In addition to VEGF-A’s central role in a variety of physiological functions, its malfunction and unregulated activity in a variety of pathological functions is an important area of research as well. For example, it has recently been recognized that angiogenesis may be involved in the pathogenesis of atherosclerosis, thus implicating a possible role for VEGF-A. VEGFs and their receptors are present, and show variations in both amount and cellular distribution, in normal arteries compared to atherosclerotic arteries [55]. VEGF-A has been shown to stimulate myocardial perfusion, and safety of VEGF-A gene-therapy has been established. Intracoronary delivery of VEGF-A gene therapy in patients with artery disease proved to be safe after eight years [56].

The pathological role of VEGF-A is perhaps best understood in the context of human tumor angiogenesis and neoplastic pathophysiology. Vasculogenesis is critical to tumor growth and metastasis, and is one of the hallmarks of cancer. Not surprisingly, numerous studies point to VEGF’s unprecedented role in tumor angiogenesis and growth. For one, VEGF-A mRNA has been found, in numerous cases, to be up-regulated in a variety of human tumors, including melanoma, breast and lung cancer, and many others [57]. Similarly, individuals with tumors expressing higher levels of VEGF mRNA were shown to have lower five-year survival rates [58]. Despite the existence of several other known pro-angiogenic factors, the importance of VEGF-A is confirmed by the efficacy of a number of different anti-VEGF-A treatments, including monoclonal antibodies and small, signal-inhibiting molecules, on hampering tumor angiogenesis and metastasis [50,59–61].

Targeting of VEGF-B also inhibits pathological angiogenesis, particularly in the eye, inhibiting both choroidal and retinal neovascularization [51] thereby offering therapeutic opportunities to treat a number of important ocular diseases. VEGF-A has also been linked to the pathogenic neovascularization of various other diseases, including inflammation and brain edema, and recent studies suggest that hyperplasia and hypervascularity, symptoms of polycystic ovary syndrome, may first arise via VEGF-A-mediated pathways [24].

Of particular importance to this paper, is research that demonstrates involvement of VEGF-A in neurological conditions, especially PD. VEGF-A, VEGF-B, VEGFR1 and VEGFR2 are all normally expressed in neurons, and astrocytes have expression induced by certain disease states [62,63]. VEGF-A has been shown to be upregulated in the substantia nigra in post-mortem brains from PD patients [64]. In a rat midbrain culture model, of PD upregulation of VEGF-B after challenge with the neurotoxin rotenone was evident [65]. VEGF-B is expressed in all neurons, including TH-positive dopaminergic neurons, in rat midbrain cultures (Falk *et al.*, unpublished results). Levels of VEGF-B have not yet been investigated in PD patients. Both VEGF-A and VEGF-B as well as their receptors have been shown to be upregulated in focal cortical dysplasia type IIB from patients with intractable epilepsy. Whereas VEGF-A was upregulated to a similar degree in neurons as well as in astrocytes, VEGF-B upregulation was found predominantly in neurons in focal cortical dysplasia type IIB [62]. Recently VEGF-A receptor-mediated response via VEGFR1 in microglia has been shown to be an integral chemotactic component in Alzheimer’s disease pathology [66]. In cultured motor neurons pretreatment with VEGF-A leads to upregulation of the glutamate receptor subunit GluR2, making them less vulnerable to AMPA receptor mediated excitotoxicity [67], a process that has been implicated to contribute to motor neuron degeneration in amyotrophic lateral sclerosis (ALS).

### 3.5. VEGFs, Neuroprotection, and Parkinson’s Disease

It has long been recognized that growth factors are involved in potentially important neuroprotective processes via direct and indirect mechanisms alike [68]. GDNF and BDNF, for example, have each been implicated in the protection of dopaminergic (DA) neurons in a direct fashion [10,68,69]. On the other hand, factors such as epidermal growth factor and fibroblast growth factor 2 have been shown to provide dopaminergic neuroprotective effects via indirect mechanisms [70]. VEGF-A’s function as a potentially therapeutic agent in the treatment of PD is supported by studies that suggest VEGF-A’s involvement in enhancement of DA neuron survival by both direct and indirect mechanisms [71,72]. VEGF-A has been shown to promote glial proliferation, and provide neuroprotective effects in response to ischemic insults [53,73]. The findings presented in the research described above led Yasuhara *et al.* to investigate the neuroprotective capacity of VEGF-A both *in vitro* and *in vivo* in model systems of Parkinson’s disease, the dose-dependent relationship between VEGF-A and neuroprotection, and the neurorescue effects of VEGF-A on *in vitro* and *in vivo* models of PD [74–76].

Initial findings show that VEGF-A has a strong neuroprotective effect on 6-hydroxydopamine (6-OHDA) treated DA neurons *in vitro* [74]. Similarly, encapsulated VEGF-A-secreting cells are shown to display a significant neuroprotective effect when implanted unilaterally into the striatum of adult unilateral-6-OHDA-lesioned PD rats [74]. Results suggest that these neuroprotective effects are dose-dependent, with low-dose administration of VEGF-A displaying more neuroprotective effects on DA neurons than high dose administration [75]. High-dose administration of VEGF-A is thought to be associated with a number of unwanted side effects, which are further discussed below.

A subsequent experiment by Yasuhara *et al.* first revealed the neurorescue effects of VEGF-A on DA neurons *in vitro* and *in vivo*. VEGF-A was administered *in vitro* via a single dose method, while *in vivo* models were subjected to continuous infusion via implanted capsules containing VEGF-A-secreting cells. In both models, VEGF-A demonstrated neurorescue effects when administered shortly after 6-OHDA-induced loss of DA neurons, while delayed treatments show little or no neurorescue effects [76].

The neuroprotective function of VEGF-A is believed to result indirectly by such functions as promoting angiogenesis and increasing glial proliferation, directly via competitively binding receptors specific to semaphorin3A, a chemorepellent known for inducing neuronal apoptosis, or both [74,75]. In all three experiments, cell survival was concomitant with increased angiogenesis and proliferation of glia.

The findings above support prolonged, low dose VEGF-A administration via encapsulated cell grafts in the treatment of PD. Encapsulated cell grafts are a particularly attractive method of treatment for a number of reasons. VEGF-A’s short half-life [77], for example, all but eliminates the efficacy of a single-dose administration. Likewise, the angiogenic properties of VEGF-A, the prototypic angiogenic factor, can prove detrimental at too high a level of administration. For example, high levels of VEGF-A can lead to unwanted side effects such as an increase in vessel density and subsequent brain edema [78,79]. Complications observed following high-doses of VEGF-A could be mitigated by the use of capsule implantation.

Other isoforms of VEGF may be better suited for use in neurodegenerative diseases. VEGF-B, which exists in two alternatively-spliced isoforms [80] has been of particular interest in the last few years. In 2008, two publications documenting VEGF-B’s neuroprotective potential were published–the first pertaining to VEGF-B_167_, the second pertaining to VEGF-B_186_ [63,81]. VEGF-B’s biological functions are poorly understood, and its role as a pro-angiogenic factor is debatable. Some studies have reported angiogenic activity [82,83], but several others have not [84–86]. The vascular survival activity of VEGF-B was achieved by the regulation of many vascular pro-survival genes via both VEGFR1 and NRP-1 [51]. With regard to neuroprotective activity, VEGF-B_167_ was shown to promote neuronal survival in the retina and the brain via downregulation of the BH3-only proteins, critical regulators in initiating apoptosis, in *in vivo* and *in vitro* models alike [81], without affecting angiogenesis. Consequently, VEGF-B_167_ was shown to have a safety profile as a neuroprotective survival molecule that is distinct from that of VEGF-A in that it exhibits few if any unwanted neurological side effects commonly associated with VEGF-A [81].

Exogenous VEGF-B_167_ and VEGF-B_186_ both have a neuroprotective effect in a rat midbrain culture model of PD after challenge with the neurotoxin rotenone [65] indicating a disease-modifying potential that needs to be further explored *in vivo*. Similarly, VEGF-B_186_ was shown to act as a neuroprotective factor for motor neurons in both *in vivo* and *in vitro* models [63]. Both experiments showed that the respective VEGF-B isoforms acted through VEGFR1, in contrast with the VEGFR2-mediated activity of VEGF-A, and were safer than VEGF-A, not causing vessel growth or blood-brain barrier leakiness at comparable doses. Given this data, it would appear as if the VEGF-B isoforms are the first of the VEGF family to confer a potent antiapoptotic effect in the absence of general angiogenic activity. These qualities support the further study of both VEGF-B isoforms for a number of neurodegenerative diseases, including PD. The VEGF-B_186_ isoform binds less to the extracellular matrix, is more diffusible than VEGF-B_167_ *in vivo* [63,80], and therefore may have a greater therapeutic potential. Preliminary results from our laboratory indeed provide initial evidence that VEGF-B_186_ is neuroprotective in an *in vivo* rat 6-OHDA-model of PD [87].

## 4. PEDF: A Widely Expressed, Pleiotropic Molecule with Anti-angiogenic, Anti-tumorigenic, and Neuroprotective Potential

As mentioned above, much of what we know about VEGF-A has stemmed from research surrounding tumor angiogenesis and neoplastic pathophysiology. Not surprisingly, research on tumorigenesis has inevitably spurred investigations that explore ways to counteract the development and progression of cancer by utilizing the body’s natural defenses [88]. Identified just four years after VEGF-A, Pigment epithelium-derived factor (PEDF) has proven to be a multifaceted protein that exhibits potent anti-angiogenic and anti-cancer activities, as well as broadly acting neurotrophic effects [88,89]. The cancer cell apoptotic pathways that PEDF interacts with have recently been reviewed in great detail [90].

PEDF was first identified in 1987 by Tombran-Tink and Johnson, who isolated the protein from retinal pigment epithelial (RPE) cell-conditioned media. Later experiments revealed that PEDF was effective in inducing differentiation of Y79 retinoblastoma cells from an actively growing cell line into non-proliferating cells with a neuronal morphology that exhibited an increase in neurite outgrowth [91], revealing PEDF’s role as a neurotrophic factor. It was later discovered that PEDF also had potent anti-angiogenic activity in its ability to inhibit the migration of endothelial cells (ECs) in a dose-dependent fashion *in vitro*. This activity was far greater than any known endogenously-produced factor, and was observed even in the presence of pro-angiogenic factors, including VEGF-A [92]. This particular aspect of PEDF’s activity suggests its intimate association with pro-angiogenic factors such as VEGF-A, as further discussed below. Since its initial isolation from fetal RPE cells, much of the research surrounding PEDF has involved the retina [93], although it is also widely present throughout the nervous system and non-neural tissues alike [94]. Presented below is an overview of PEDF’s structure, function, and neuroprotective activity, as well as its potential role in the treatment of PD.

### 4.1. PEDF Structure

PEDF is a member of the serine protease inhibitor (serpin) superfamily of proteins, although it does not function as a protein inhibitor [95,96]. The PEDF gene consists of eight exons interspersed with seven introns [97]. The gene codes for a 418-amino acid polypeptide that contains an N-terminal hydrophobic signal peptide sequence characteristic of secreted proteins [53], an N-glycosylation site at Asn285 [95], and a homologous serpin reactive center loop (RCL) located at the C-terminal end of the polypeptide chain [95]. The mature gene product is secreted in its glycosylated form, which increases the overall weight of the protein from 46.3 kDa to 50 kDa [96]. When PEDF folds into its globular form, PEDF’s RCL remains exposed, but PEDF is unique as a member of the serpin superfamily of proteins in that its RCL lacks the serine protease inhibitory activity that it typically confers to other serpins [98].

Asymmetric charge distribution along the polypeptide creates a final structure with basic residues and acidic residues localized on either side of the molecule [98]. These different regions of the PEDF glycoprotein allow it to interact with various components of the ECM. The region of the protein rich in the basic amino acids Lys and Arg mediates low-affinity interactions between heparin and other glycosaminoglycans [99], whereas Asp and Glu-rich regions of the N-terminal portion of PEDF facilitate binding to type I collagen and type III collagen with high and low affinity, respectively [100,101]. The particular usefulness of having PEDF bound in the ECM is not entirely understood, but could relate to its ability to be stored for release, or perhaps its positioning for interacting with a cell surface receptor.

A number of peptide regions are particularly important in PEDF’s various activities. Its anti-angiogenic and tumor-cell apoptosis activities, for example, both seem to correspond to a 44-residue fragment (residues 78–121) [102,103]. In contrast, PEDF’s neurotrophic and cell differentiation activities have been shown to correspond to a 34-residue fragment (residues 44–77) [98,103]. Furthermore, the negatively-charged collagen binding motif mentioned above has been reported as essential to PEDF’s anti-angiogenic properties [104], whereas various states of phosphorylation at Ser24, Ser114, and Ser227 have been shown to mediate PEDF’s neurotrophic and anti-angiogenic activities alike [88,103]. These activities are hallmarks of PEDF function, and are discussed in further detail below.

### 4.2. PEDF Receptors and Cell Signaling

**Figure 2:**
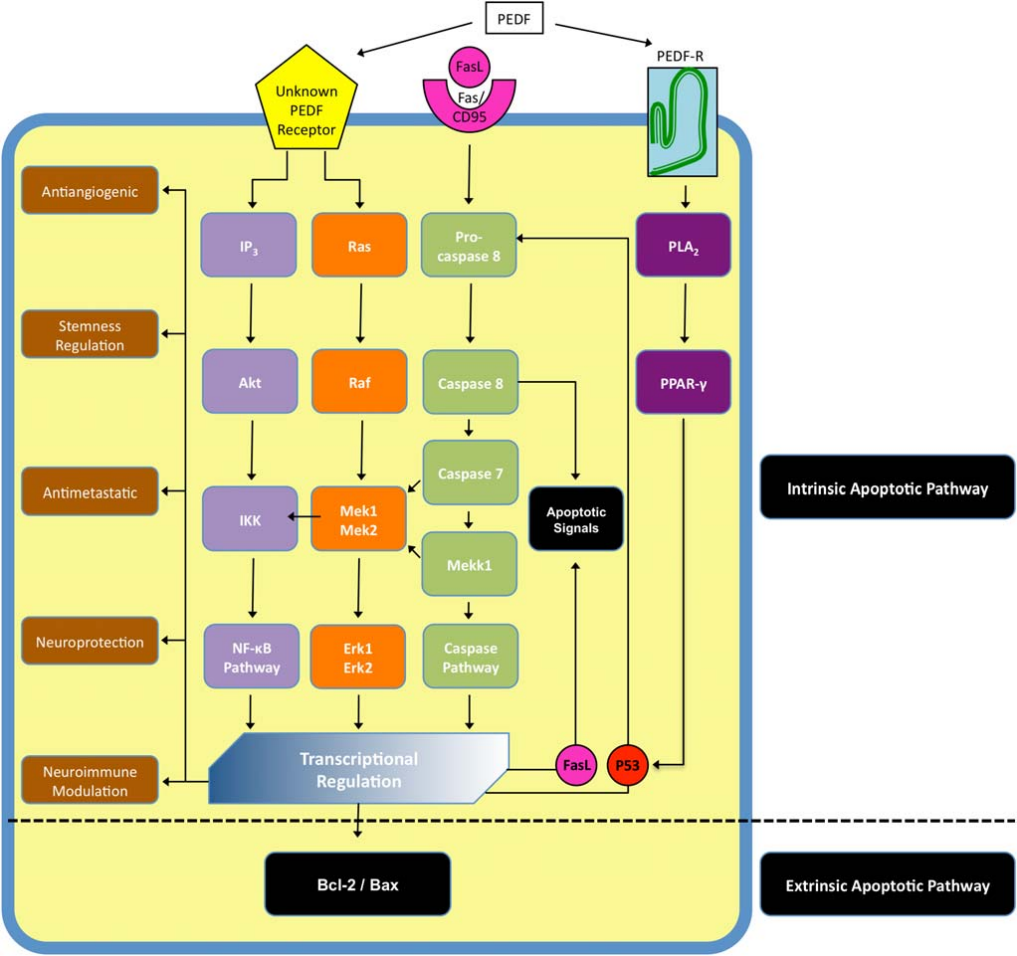
**Schematic of pathways activated by PEDF**. By binding to more than one receptor, one of which has been identified, PEDF activates several intracellular signaling cascades that have extensive crosstalk and regulate a host of functions, including both the extrinsic and the intrinsic apoptotic pathways. (FasL, Fas ligand; CD95, Fas receptor; Ras, ras superfamily GTPases; Raf, RAF proto-oncogene serine/threonine-protein kinase; IKK, IκB kinase; Mekk1, also known as mitogen-activated protein kinase kinase kinase 1; Mek, also known as dual specificity mitogen-activated protein kinase kinase; Erk, extracellular-signal-regulated kinase; P53, tumor protein 53; PLA_2_, phosospholipase A2; PPAR-γ, peroxisome proliferator-activated receptor γ; Bcl, B-cell lymphoma 2; Bax, Bcl-2–associated X protein).

Unlike VEGF-A, the receptors with which PEDF interacts are poorly understood. The transduction pathways by which it effects cellular change, while better understood than its receptors, continue to be researched and characterized. As outlined below it likely interacts with more then one cell surface receptors resulting in a signaling cascade wherein the initial signal is transduced via several intracellular signaling pathways (Figure 2).

Numerous experiments reveal PEDF to have a high affinity for binding sites on retinoblastoma cells, cerebellar granule cells, motor neurons and ECs, pointing to PEDF’s interaction with an unknown receptor or receptor type [99,102]. Recently a phospholipase-linked membrane protein has been identified as a possible receptor for PEDF [105]. The PEDF-R (Figure 2) has putative transmembrane, intracellular and extracellular regions, and a phospholipase domain. It is a member of the calcium-independent PLA_2_/nutrin/patatin-like phopholipase domain-containing 2 (PNPLA2) family of proteins with triglyceride lipase and acylglycerol transacylase activity. PEDF binding stimulates the enzymatic phospholipase A_2_ activity of this PEDF-R. The downstream pathways of this receptor were then further elucidated by Ho *et al.* who proved a sequential activation of PLA_2_, peroxisome proliferator-activated receptor gamma (PPAR-γ) and p53 as the mechanism of PEDF-induced apoptosis (Figure 2) in endothelial cells [106].

Blockade of this PEDF receptor did not inhibit another recently discovered activity of PEDF though, namely its modulation of neurosphere formation. PEDF does modulate Notch-dependent stemness in the adult subependymal zone via an unidentified receptor. Besides the Notch pathway, activation of the nuclear factor-κB (NFκB) is required. This indicates that PEDF and Notch cooperate to regulate self-renewal [107].

Evidence of PEDF’s neuroprotective capacity has been elucidated in cerebellar granule cells in particular. In these cells, binding of PEDF has also been shown to stimulate the activation of NF-κB signaling cascade, wherein terminal activation of the NF-κB protein complex allows NF-κB to act as a transcription factor that induces the expression of genes critical to neuronal protection and survival, including BDNF and GDNF [108].

In contrast, PEDF produces anti-angiogenic activity by a different, two-fold mechanism (Figure 2). The first involves induction of the Fas ligand (FasL)—Fas/CD95 (FasL receptor) cell death pathway. PEDF increases synthesis of FasL, resulting in an increase of the pathway initiated by its binding of the Fas/CD95 receptor, resulting in EC apoptosis—in this case ECs to be utilized in neovascularization [108]. The second involves a direct competition with VEGF-A’s actions as a pro-angiogenic factor [108]. The critical interaction between VEGF-A’s pro-angiogenic function and PEDF’s anti-angiogenic function and the balance of each is discussed in greater detail below.

In addition to the cancer-relevant PEDF research mentioned earlier, a recent finding is interesting given the known increase of melanoma risk in PD patients [109]. A very recent publication pointed to high PEDF expression in melanocytes that is lost during malignant progression of melanomas. This loss of PEDF enables migration, invasion and metastatic spread of human melanoma [110]. Possible regulatory mechanisms underlying the changes in PEDF expression during transformation and malignant progression of melanoma are presently unknown, although there are several likely candidates including oncogenes and tumor suppressor genes [90]. For example PEDF is a direct target of p53 family members in colorectal cancer cell lines [111].

### 4.3. PEDF, Biological Function, Neuroprotection, and Parkinson’s Disease

PEDF’s involvement in cell survival and cell death discussed in the above two sections emphasize PEDF’s distinction as a growth factor that is both neuroprotective, and a highly potent endogenous modulator of angiogenesis. It shares these traits in common with VEGF-A. Both VEGF-A and PEDF are neuroprotective, making them interesting targets for the treatment of Parkinson’s disease, but they have opposing functions on vascular systems. These opposing actions are particularly prominent in the eye, where many studies have shown how angiogenic homeostasis is maintained by a careful balance between angiogenic stimulators, like VEGF-A, and angiogenic inhibitors like PEDF [112–114]. The existence of this balance in the eye suggests that PEDF may normally be involved in neovascularization and vascular maintenance in other parts of the body, perhaps opposing the action of VEGF-A discussed in the previous section. This possibility is further reinforced by numerous observations that PEDF mRNA is widely expressed throughout the body in non-neural tissues such as skeletal muscle, bone, and liver [94,97].

The initial studies characterized PEDF by its tendency to promote cell differentiation, as evidenced by its neurotrophic effects on retinoblastoma cells [89,91]. While the role of PEDF in the eye has been the focus of most studies, the wide distribution of PEDF mRNA in the CNS has led to multiple studies on its role as a neurotrophic and neuroprotective factor in non-retinal neurons [93,102,115,116]. For example, administrations of low concentrations of PEDF improved the survival of cerebellar granule cells in culture in several studies [108,117,118]. PEDF’s protective activity has been observed in glutamate-induced models of apoptosis in hippocampal and spinal motor neurons as well [115,116]. Similarly, prompted by studies that showed the ability of a serpin related to PEDF to promote the *in vivo* survival and growth of motor neurons, a study by Houenou *et al.* provided evidence of PEDF’s function in neuron protection *in vivo.* The study subjected neonatal mice to sciatic nerve sectioning, which typically led to the death of roughly half the motor neurons in the mice, followed by atrophy of many of the surviving cells. Treatment with varying amounts of small quantities of PEDF leads to as much as a 57% decrease in motor neuron death and prevented atrophy of any surviving neurons [119]. Crawford *et al.* have shown that PEDF’s dual neuroprotective and apoptotic activity may allow for a positive feedback mechanism in the regression of neuroblastomas. Their study showed that injection of PEDF into tumors has multiple activities. It not only promotes the differentiation of primitive neuroblastoma cells, but also promotes Schwann cell survival and mitogenesis [120]. Moreover, PEDF was observed to increase Schwann cell secretion of “antineuroblastoma” agents, including PEDF, which could act back on surrounding neuroblastoma in a positive feedback loop [85].

Studies that monitor PEDF levels in response to progressive neurological diseases like Amyotrophic Lateral Sclerosis (ALS) show that there is a significant upregulation of PEDF in the cerebrospinal fluid (CSF) of patients affected by the disease [121]. These types of results, combined with observations that highlight the neuroprotective effects of supplementation with PEDF, suggest that its secretion by tissues may reflect their attempt to limit neuronal damage. Findings such as these, when combined with other studies which show that PEDF expression declines with aging, have led some to propose that the increasing incidence of neurodegenerative diseases with age may be linked to a decrease in PEDF expression throughout the body [93].

Interestingly, retinal pigment epithelial cells (RPE), from which PEDF was first isolated and characterized, have recently been suggested to possess a dual-mechanism of action for the treatment of PD’s symptoms and underlying causes [122]. Since levodopa is an intermediate in the melanin biosynthetic pathway active in RPE cells, evidence suggesting that striatal implantation of RPE cells could provide a continuous source of levodopa for neuronal conversion to dopamine has been presented in numerous studies [9,123]. In 2006, McKay *et al*. found that RPE cell-conditioned media contains multiple neurotrophic factors that increase neurite outgrowth in cultured dopaminergic neurons [122]. Their findings indicated that RPE cells are capable of conferring a robust neurotrophic effect on dopaminergic neurons of the midbrain, especially “differentiated” RPE-conditioned media (RPE-cm), which exhibited the most potent neurotrophic effect [122]. Differentiation refers to culture conditions that more closely emulate the properties of adult RPE found *in vivo* such as dense pigmentation and polygonal morphology. It was observed, for example, that differentiated RPE-cm expressed significantly higher levels of PEDF than undifferentiated RPE-cm cells.

McKay *et al.* found that the majority of the neurotrophic effect was attributed to PEDF, although the action of other neurotrophic factors was imputed to by residual neurotrophic activity following PEDF depletion. Conditioned media from differentiated RPE cultures was assayed after depletion of PEDF for effects on neurite outgrowth. The relative depletion of PEDF was verified by ELISA [122]. The level of PEDF was reduced by over 80%, and the neurotrophic effect was diminished by ≈50%. This demonstrates a strong neurotrophic effect of PEDF elaborated from RPE. Interestingly, these differentiated RPE do also produce substantial quantities of VEGF-A, measured by ELISA (Falk *et al.*, unpublished results). To complement these findings, a later report by Ming *et al.* [124] concluded that more traditional growth factors, such as GDNF and BDNF, were also elaborated by RPE cells, and in this paradigm accounted for a significant portion of the neurotrophic effect. Together this points to PEDF as one of the important but not sole mediators of neurotrophic influence of RPE. The research was recently extended to show that PEDF is not only neurotrophic but also neuroprotective in both a 6-OHDA and a rotenone primary midbrain culture model of PD [125].

The findings of these studies have strong and timely implications for the future of therapeutic studies for PD. A recent Phase II trial of RPE cell transplantation (STEPS trial) failed to show improvement over sham-operated controls [126]. However, this trial had a number of short-comings that should be addressed in future trials of RPE cell transplantation. For example, prior studies utilizing RPE were designed *a priori* to test the potential for dopamine replacement. Since a natural function of the RPE is to provide trophic support for neural cells, we would argue that RPE transplantation can provide, in physiological doses, a cocktail of endogenously produced trophic factors rather than a single, exogenously introduced agent studied in other neurotrophic strategies. Therefore first, it will be critically important to ensure that the RPE cells prepared for implantation are screened for there full neurotrophic potential, including the level of PEDF production. Second, the clinical endpoint of the trial should include an objective measure of neuroprotection or modification of the underlying disease process. This latter point is particularly true in light of the particularly large placebo response noted in the preliminary reports from this trial. While future studies will be determined to truly evaluate the potential of the RPE cell platform, other delivery methods such as direct protein infusion or gene therapy approaches to increasing levels of PEDF remain a viable therapeutic target.

## 5. The Yin and Yang of VEGF and PEDF

As alluded to throughout this review, the function of VEGF-A is intimately associated with that of PEDF. In their seminal work that revealed PEDF as an anti-angiogenic factor, Dawson *et al.* revealed that in oxygen-deficient conditions in culture, cell secretion of VEGF-A was increased while secretion of PEDF was decreased [92]. This makes sense, as oxygen deprivation is known to stimulate angiogenesis, as is commonly observed in the progression of cancer. Given VEGF-A’s history as a pro-angiogenic factor, these results suggest that shifts in angiogenic activity are dependent upon a critical balance between pro-angiogenic factors such as VEGF-A, and anti-angiogenic factors like PEDF.

Questions surrounding the existence of such a balance led to a number of studies that, in turn, reinforced the notion of a critical balance between VEGF-A and PEDF in a variety of ocular diseases. The first example of this was published in 2001 by Gao *et al*., who demonstrated that PEDF levels are negatively correlated with pathological retinal neovascularization, while VEGF-A levels were positively correlated [112]. The existence of a critical VEGF-A/PEDF balance was further reinforced by findings that showed the time course of PEDF down-regulation to be coincident with an increase in VEGF-A expression. The lowest PEDF levels not only coincided with the highest VEGF-A levels, but also with the development and progression of retinal neovascularization [112].

The balance between VEGF-A and PEDF have been implicated in the mechanism of other ocular diseases, including macular edema and macular degeneration [113,114,127–129]. The effects of this balance on retinal angiogenesis have been shown in these cases to be of significant importance to the survival of retinal neurons, which could degenerate in an environment permissive to the growth of permeable blood vessels.

Mechanistically, one way PEDF inhibits VEGF-A-induced angiogenesis is by disrupting the PI3K/Akt signaling pathway [130]. Further insight into mechanisms involved in the balancing of VEGF-A and PEDF is provided by data showing that hypoxia and VEGF-A can down-regulate PEDF through proteolytic degradation. PEDF is a substrate for the matrix metalloproteinases MMP-2 and MMP-9 [131]. These results reveal a posttranslational mechanism for down-regulating PEDF, and provide an explanation for hypoxia-induced increases in VEGF-A/PEDF ratios, in angiogenesis and/or in neuronal death. Interestingly, the neurotoxin rotenone not only upregulates VEGF-B but also MMP- 9 in rat midbrain cultures [65] indicating another point of crosstalk. A recent study also suggests that PEDF can decrease mitochondria-derived reactive oxygen species generation, and subsequently down-regulate VEGF-A expression, possibly through inhibiting janus kinase 2/signal transducer and activator of transcription 3 (JAK2/STAT3) activation [132].

Questions surrounding the ratio of VEGF-A to PEDF and neurons other than retinal neurons recently gave rise to a study involving a model of PD [133]. In the study, Yasuda *et al.*, recognizing PEDF’s protective effects on various neuronal populations, measured the levels of PEDF in the striatum and substantia nigra of PD patients, as well as in mouse models of PD, to characterize its role in the pathophysiology of the disease. This study also monitored the striatal levels of VEGF-A relative to PEDF levels following an insult to the dopaminergic pathway [133]. The results from the mouse model suggest that acute damage to dopaminergic neurons induces a rise in PEDF levels in the CNS, which supports the hypotheses regarding PEDF’s actions as a natural neuroprotective factor. Likewise, a moderate, albeit non-significant, increase in PEDF levels was observed in the striatum of PD patients. The most intriguing finding, however, was a significantly positive correlation in the striatal levels of PEDF and VEGF-A in PD patients [133], which suggested a possible concerted neurotrophic effect by these factors. The recent evidence for changes in VEGF-B levels in a toxin-model of PD [65] suggests VEGF-B levels in PD patients should be investigated as well, in order to confirm VEGF-B as another natural neurotrophic factor.

While PEDF and VEGF-A seem to work in opposition with respect to vascularization, their relationship with respect to neuronal survival is not so clear cut. Both factors have positive effects on neuronal survival and may work in concert here rather than in opposition. Nevertheless, the possibility of a link between them is consistent with their intertwined actions elsewhere.

## 6. Conclusion

The complex but promising correlation between the functions of VEGF’s and PEDF warrants further investigation into their capacities as therapeutic agents in the treatment of PD, not only individually, but also in concert. VEGF-A and PEDF act in opposition in vascular beds, which have been well-studied in the eye and have significant implications for the survival of retinal neurons. A similar opposing effect is seen in other tissues, particularly with regard to cell proliferation. In the brain, however, VEGF-A, VEGF-B, and PEDF all share potent neuroprotective effects that are likely separate from the vascular effects seen in the periphery. While the neuroprotective effects work in concert in the brain, their regulation in neurodegenerative diseases such as Parkinson’s disease may be linked in a reciprocal fashion [133]. Recent studies of VEGF-B suggest that this isoform is particularly deserving of further study since it appears to have a natural role as a response to neurotoxins and potentially exerts a neuroprotective effect in the brain without increasing vascular permeability. While there is abundant information regarding the mechanisms of action for PEDF and VEGF-A, the pathways that are activated by VEGF-B require further delineation. Future work should include the possibility of an intrinsic link between the regulation of these factors and potential crosstalk in their downstream signaling systems.

An expanding population of the elderly that are susceptible to neurodegenerative diseases demands immediate action be taken to explore novel agents for slowing disease progression. In the case of Parkinson’s disease, there is relatively selective vulnerability of dopaminergic neurons in the midbrain that may be amenable to disease modification using neurotrophic growth factors. The recent work discussed in this review, puts the VEGF’s and PEDF on the list of promising neurotrophic growth factors for PD. Recent research on VEGF-A, VEGF-B and PEDF has revealed neuroprotective properties of these proteins with respect to dopaminergic neurons as well as other selected neuronal populations. Given the possible links between these factors, future research should investigate if cocktails made of growth factors that work in concert can surpass the positive effect of single growth factors in animal models of PD. RPE cell-transplantation for Parkinson’s disease has been proven to be a viable platform in Phase II trials but was not effective as a source for dopamine replacement. Given that these cells produce a cocktail of growth factors including both PEDF and VEGF-A the potential neuroprotective properties of RPE cell transplantation should not be dismissed. With neuroprotection, disease modification, and neurorestoration as the most critical objectives of PD research, these proteins first studied for their effect of vascularization are likely to provide an unexpected but welcome effect on neuronal survival.
